# Prevalence and Risk Factors of Anemia in Patients After Bariatric Surgery in Qassim Region, King Fahad Specialist Hospital

**DOI:** 10.7759/cureus.40131

**Published:** 2023-06-08

**Authors:** Bandar Saad Assakran, Renad Khalid, Thekra Bennasser, Maha Alsaif, Watin Alsawyan, Haifa Alsaleem, Ahlam Alsalhi

**Affiliations:** 1 General Surgery, King Fahad Specialist Hospital, Buraydah, SAU; 2 College of Medicine, Qassim University, Buraydah, SAU; 3 Family Medicine, Primary Health Care Al Muntazah, Buraydah, SAU

**Keywords:** sleeve gastrectomy, obesity, bmi, anemia, bariatric surgery

## Abstract

Introduction

There is a high prevalence of obesity among the Saudi population. Anemia due to iron deficiency or an inflammatory state is often associated with obesity. Multiple nutritional deficiencies are associated with bariatric surgeries, with anemia being one of the commonest causes.

Aim

This study aimed to evaluate the prevalence of anemia after bariatric surgery among patients in the Qassim Region, Saudi Arabia.

Patients and methods

This retrospective cohort study was conducted at King Fahad Specialist Hospital Al-Qassim (Buraydah), Saudi Arabia. We reviewed data from patients' records who underwent bariatric surgeries from January 2018 to January 2021. By using a structured data collection form, we collected data such as demographic variables, surgery perioperative-related data, postoperative complications and interventions, type of transfusion required after surgery, postoperative medications and/or supplements and duration, and blood count indices.

Results

Of the 520 patients who underwent bariatric surgery, 61% were females, and 31.7% were aged between 26 to 35 years old. The most prominent type of bariatric surgery was sleeve gastrectomy (97.1%). The prevalence of anemia among patients who underwent bariatric surgery was 28.1%. Independent risk factors for anemia were female gender, microcytic red blood cells, and low normal hematocrit and hemoglobin (Hgb) levels. It is interesting to know that sleeve gastrectomy and elevated BMI levels are considered to be the protective factors for developing anemia postoperatively.

Conclusion

There was a high prevalence of anemia among bariatric patients postoperatively. Female gender with decreasing hematocrit and hemoglobin levels after the surgery might be more at the receiving end for developing anemia than the other patients. Further longitudinal studies are needed to establish the prevalence and risk factors for developing anemia among bariatric surgery patients.

## Introduction

Obesity is a chronic disease with high prevalence in Saudi Arabia. The overall rate of obesity in the Kingdom of Saudi Arabia (KSA) is 33.7% and estimated to reach 59.5% in 2022. Obesity is a chronic disease which may lead to serious complications, such as diabetes mellitus, cardiovascular disease, osteoarthritis, dyslipidemia, and psychological disability. Bariatric surgery is an effective method for weight reduction and it’s a reasonable option considered for significant weight loss and reduction in comorbidities but there’re various types of nutritional deficiencies that may happen after Bariatric surgery which increase the risk of anemia [1].

Obesity is associated with multiple factors, not only genetic but physiological factors, psychological issues, and environmental variables, both physical and social, affecting individuals of all ages and regions. High prevalence rates for obesity and overweight are seen in Saudi Arabia due to the increasing westernization over the last few decades, leading to unhealthy eating, sedentary lifestyles, and weight gain [2].

Anemia due to iron deficiency or inflammatory state is often associated with obesity. Bariatric surgery is responsible for increasing iron deficiency, but weight loss decreases the inflammatory state associated with obesity. Anemia is very frequent in severely obese patients and must be investigated both before and after bariatric surgery. The cause of anemia must be determined in order to use the best treatment available [3].

Iron is an essential element of hemoglobin and it participates in many processes including oxygen transport, deoxyribonucleic acid (DNA) synthesis, and electron transport [4]. Any disruption in its levels may lead to health problems like anemia. Iron deficiency anemia is defined as the state at which hemoglobin (Hb) and hematocrit (Hct) levels fall under indicated referenced cut-off points depending on age and gender [5].

Iron deficiency is a common consequence of bariatric surgery and frequently leads to anemia [6]. The risk of developing nutritional deficiencies is increased after each of the bariatric surgeries. These deficiencies supposedly cause a wide range of long-term complications, including nutritional, metabolic and neurological ones [7].

These deficiencies develop as a consequence of the alterations in the gastrointestinal anatomical architecture and the associated changes in the physiology of the gastrointestinal tract, the risk of developing iron deficiency as it impairs the normal physiological function of tissues such as blood, brain and muscles. Many factors contribute to the development of iron deficiency after bariatric surgery such as reduced iron intake, reduced secretion of hydrochloric acid and a reduction in the surface area for absorption [8].

Monitoring the iron (Fe) status before bariatric surgery is of crucial importance. In general, in symptoms including weakness, fatigue, irritability, and apathy, the iron deficiency diagnosis should always be considered, even if there is no anemia [9].

The B complex vitamins are integral to the synthesis of neurotransmitters and proper functioning of the central nervous system and any defect may lead to serious problems to the individual [10], because vitamin B12 storage depletion takes many years and high-dose oral vitamin B12 supplementation is very effective for prevention of this micronutrient deficiency [11].

## Materials and methods

Study design, setting and duration

This is a hospital-based retrospective descriptive study conducted in King Fahad Specialist Hospital in Qassim, Buraydah, Saudi Arabia. It included all bariatric surgeries from January 2018 to January 2021. The data was obtained from patients' lab results pre- and post-surgery.

Sample size

All the records of 520 patients who underwent bariatric surgeries, from January 2018 to January 2021, were obtained from King Fahad Specialist Hospital (Buraydah) Saudi Arabia after the application of inclusion and exclusion criteria.

Sampling technique (with inclusion and exclusion criteria)

A convenience sampling method was used.

Inclusion Criteria

Patients (>16 years) who underwent bariatric surgery at King Fahad Specialist Hospital between January 2018 and January 2021 and had completed six months or more of post-surgery follow-up.

Exclusion Criteria

Patients aged <16 years, patients with already established gastrointestinal, renal and/or hematologic diseases, pregnant patients, patients with incomplete medical records, patients who previously underwent bariatric surgery, and patients with less than six months postoperative follow-up.

Data collection methods

Data extraction form was designed according to the objectives of the study. By using the patients' records, the following information was extracted: demographic data (age, gender, nationality), bariatric surgery perioperative-related data (anthropometric measurements), postoperative interventions, type of transfusion required after surgery, postoperative medications and/or supplements and duration, and laboratory tests performed at the time of surgery and at the last visit, including blood count indices, serum vitamin B12 level.

Data management and analysis plan

Data Management

Reviewing the incidence of anemia in patients who underwent bariatric surgery between January 2018 and January 2021, at King Fahad Specialist Hospital. Types of anemia and its association with different types of bariatric surgery will be represented, and we will assess demographic data associated with anemia as well.

Analysis Plan

Variables were coded, then analyzed by using Statistical Package for the Social Sciences (SPSS). The results were featured with the use of a simple percentage (%). A probability level (p-value) of 0.05 or less was used to point out statistical significance.

Statistical analysis

For the descriptive statistics, the mean and standard deviation were used for metric variables, whereas frequencies and proportions (%) were given for categorical variables. The association between anemia and the demographic and clinical characteristics of the patients who underwent bariatric surgery was calculated using the Chi-square test. Based on the significant results, multivariate regression analysis was subsequently performed to determine the significant independent factors associated with anemia post-bariatric surgery. Paired sample t-test was also conducted to determine the differences in the mean values of BMI and complete blood count before and after bariatric surgery. All statistical analyses were computed using the software program Statistical Package for the Social Sciences (SPSS) version 26 (IBM Corp., Armonk, NY). Values were considered significant with a confidence interval of 95% (p<0.05).

## Results

We analyzed 520 patients who underwent bariatric surgery. As described in Table 1, the most common age group was 26 to 35 years old (31.7%), with females being dominant (61%). The most commonly associated comorbidity was diabetes (18.7%) and hypertension (14.4%).

**Table 1:** Demographic and clinical characteristics of the patients (n=520) † Some patients have more than one comorbidity.

Study variables	N (%)
Age group	
16 - 25 years	114 (21.9%)
26 - 35 years	165 (31.7%)
36 - 45 years	150 (28.8%)
>45 years	91 (17.5%)
Gender	
Male	203 (39.0%)
Female	317 (61.0%)
Comorbidities ^†^	
None	255 (49.0%)
Diabetes	97 (18.7%)
Hypertension	75 (14.4%)
Hypothyroidism	57 (11.0%)
Asthma	45 (08.7%)
Infertility	36 (06.9%)
Rheumatic disease	10 (01.9%)
Mental disorder	08 (01.5%)
Heart disease	06 (01.2%)
Other	32 (06.2%)

In Table 2, the most prominent type of bariatric surgery was sleeve gastrectomy (97.1%). Primperan (Metoclopramide) (33.5%) was the most common medication after the surgery, and two-thirds (66.9%) took medications for at least less than 90 days. Most of the patients took multivitamins (97.7%) for approximately 90 days or more (80.8%).

**Table 2:** Surgical procedure and medications (n=520)

Variables	N (%)
Type of bariatric surgery	
Sleeve gastrectomy	505 (97.1%)
Gastric bypass	15 (02.9%)
Postop medication	
Primperan (Metoclopramide)	174 (33.5%)
Tramadol	151 (29.0%)
Omeprazole	123 (23.7%)
Esomeprazole	62 (11.9%)
Others	10 (01.9%)
Duration of medication	
<90 days	348 (66.9%)
≥90 days	172 (33.1%)
Postop supplements	
Multivitamins	508 (97.7%)
Vit B complex	07 (01.3%)
Vit D3	05 (01.0%)
Duration of supplements	
<90 days	100 (19.2%)
≥90 days	420 (80.8%)

**Figure 1:**
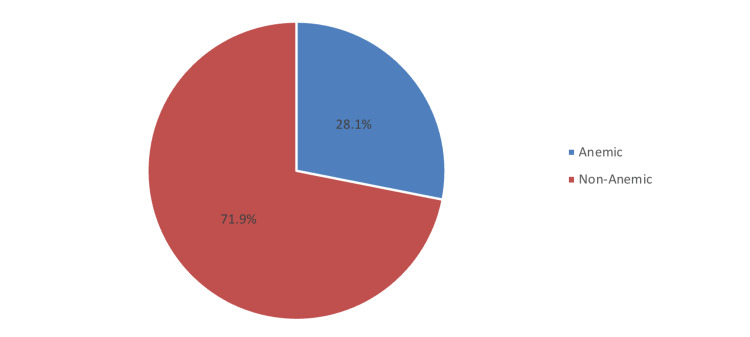
Prevalence of anemia after bariatric surgery The prevalence of patients who developed anemia postoperatively was 28.1%, while the rest were normal (71.9%).

The prevalence of patients who developed anemia postoperatively was 28.1%, while the rest were normal (71.9%) (Figure 1).

Comparing the differences in the mean values of BMI and CBC before and after bariatric surgery (Table 3), it was observed that the BMI mean values were statistically significantly lower postoperatively (p<0.001), while the mean corpuscular volume (MCV) values were statistically significantly higher after bariatric surgery (p<0.001). However, the differences in the mean values of Hgb, hematocrit, and vitamin B12 before and after the surgery did not reach statistical significance (p>0.05).

**Table 3:** Paired t-test of the BMI and the complete blood count (CBC) before and after bariatric surgery (n=520) § P-value has been calculated using Paired sample t-test. ** Significant at p<0.05 level; MCV: Mean corpuscular volume

Variables	Before Mean ± SD	After Mean ± SD	P-value ^§^
BMI	44.6 ± 6.24	26.7 ± 5.81	<0.001 **
Hemoglobin	13.3 ± 1.73	13.5 ± 2.77	0.140
Hematocrit	41.5 ± 18.8	40.9 ± 6.91	0.514
MCV	82.4 ± 7.34	85.8 ± 7.60	<0.001 **
Vit B12	365.6 ± 223.7	340.1 ± 187.1	0.160

When measuring the association between postoperative anemia and the demographic and clinical characteristics of the patients (Table 4), it was found that the prevalence of postoperative anemia was significantly more common among the female gender (p<0.001), those who underwent gastric bypass (p=0.027), those who had less than 25 kg/m^2^ post-BMI levels (p=0.050), those who had low post hematocrit level (p<0.001), or low post-Hgb level (p<0.001), those who were considered as microcytic anemia (p=0.013) and those who had low post-vitamin B12 level (p<0.001).

**Table 4:** Association between postoperative anemia and the demographic and clinical characteristics of the patients who underwent bariatric surgery (n=520) † Some patients were lost to follow-up. ‡ Patients within the normal range were excluded from the analysis. § P-value has been calculated using the Chi-square test. ** Significant at p≤0.05 level; MCV: Mean corpuscular volume

Factor	Anemia		P-value ^§^
	Anemic N (%) (n=146)	Non-anemic N (%) (n=374)	
Age group			
≤35 years	70 (47.9%)	209 (55.9%)	0.103
>35 years	76 (52.1%)	165 (44.1%)	
Gender			
Male	26 (17.8%)	177 (47.3%)	<0.001 **
Female	120 (82.2%)	197 (52.7%)	
Associated comorbidities			
No	67 (45.9%)	188 (50.3%)	0.370
Yes	79 (54.1%)	186 (49.7%)	
Type of bariatric surgery			
Sleeve gastrectomy	138 (94.5%)	367 (98.1%)	0.027 **
Gastric bypass	08 (05.5%)	07 (01.9%)	
Postop medication			
Omeprazole	29 (20.3%)	94 (25.6%)	0.526
Esomeprazole	16 (11.2%)	46 (12.5%)	
Tramadol	44 (30.8%)	107 (29.2%)	
Primperan	54 (37.8%)	120 (32.7%)	
Post BMI level ^†^			
<25 kg/m^2^	10 (33.3%)	13 (16.3%)	0.050 **
≥25 kg/m^2^	20 (66.7%)	67 (83.7%)	
Post Hematocrit level ^‡^			
Low	82 (97.6%)	17 (19.3%)	<0.001 **
Elevated	02 (02.4%)	71 (80.7%)	
Post Hgb level ^‡^			
Low	120 (99.2%)	07 (22.6%)	<0.001 **
Elevated	01 (0.80%)	24 (77.4%)	
Post MCV level ^‡^			
Microcytic	62 (98.4%)	38 (86.4%)	0.013 **
Macrocytic	01 (01.6%)	06 (13.6%)	
Post Vitamin B12 level ^†^			
Low	32 (34.4%)	0	<0.001 **
Normal	61 (65.6%)	210 (100%)	

In a multivariate regression model (Table 5), after adjustment with age and comorbidities, it was found that compared to gender males, females were predicted to increase the risk of anemia by at least 4.1 times higher (AOR=4.079; 95% CI=2.530-6.575; p<0.001). However, patients who underwent sleeve gastrectomy were predicted to decrease the risk of anemia by at least 68% compared to those who underwent gastric bypass (AOR=0.328; 95% CI=0.116-0.924; p=0.035), while patients who had BMI levels of 25 kg/m^2^ or more were also predicted to decrease the risk of anemia by at least 73% (AOR=0.274; 95% CI=0.094-0.801; p=0.018). Patients with low hematocrit levels after the surgery were predicted to increase the risk of anemia as much as 181 times higher compared to those with elevated one (AOR=181.1; 95% CI=39.5-831.7; p<0.001). Similarly, compared to patients with elevated Hgb, patients who had low Hgb levels after the surgery were more likely to have an increased risk for anemia by at least 456-fold higher (AOR=456.2; 95% CI=48.9-4256.1; p<0.001). In addition, compared to patients who were considered macrocytic, patients who were considered microcytic were at increased risk for anemia by at least 10 times higher (AOR=10.027; 95% CI=1.121-89.712; p=0.039).

**Table 5:** Multivariate regression analysis to determine the significant independent predictors of anemia post-bariatric surgery (n=520) † Some patients were lost to follow-up. ‡ Patients within the normal range were excluded from the analysis. AOR – Adjusted Odds Ratio; CI – Confidence Interval. Adjusted with age and comorbidities. ** Significant at p<0.05 level.

Factor	AOR	95% CI	P-value
Gender			
Male	Ref		
Female	4.079	2.530 – 6.575	<0.001 **
Type of bariatric surgery			
Sleeve gastrectomy	0.328	0.116 – 0.924	0.035 **
Gastric bypass	Ref		
Post BMI level ^†^			
<25 kg/m^2^	Ref		
≥25 kg/m^2^	0.274	0.094 – 0.801	0.018 **
Post Hematocrit level ^‡^			
Low	181.1	39.5 – 831.7	<0.001 **
Elevated	Ref		
Post Hgb level ^‡^			
Low	456.2	48.9 – 4256.1	<0.001 **
Elevated	Ref		
Post MCV level ^‡^			
Microcytic	10.027	1.121 – 89.712	0.039 **
Macrocytic	Ref		

## Discussion

This study investigated the prevalence of risk factors for anemia after bariatric surgery. Our findings suggest that the prevalence of anemia after bariatric surgery was high. Approximately 28.1% of our patients developed anemia postoperatively. This prevalence is almost consistent with the study done in Al Madinah, in which Alwasaidi et al. reported that 22.9% of the patients had been diagnosed with anemia post-bariatric surgery, with all being women [1].

Bariatric surgery has been the most effective solution for weight loss and comorbidities. However, despite its great benefits, this could pay some price at some point, including postoperative complications. Of all complications, nutritional deficiencies are represented with greater concerns that need extra attention [12]. There has been a report that the prevalence of anemia increased progressively during follow-ups. For example, a study by de Cleva et al. [3] found that after the Roux-en-Y gastric bypass (RYGB) surgery, 8.8% of patients had developed anemia (mild: 93.2%) and anemia due to chronic disease was 43.8%. After 24 months of follow-up, iron-deficiency anemia (IDA) increased to 72.4%, whereas a decrease in anemia due to chronic disease has been reported at 15.5%. According to the literature review by Steenackers et al. [8], the prevalence of IDA in a 10-year duration ranges from 6.6 to 22.7% after RYGB. In comparison, the prevalence of anemia among patients who underwent sleeve gastrectomy (SG) ranged between 3.6% and 52.7% [13-23]. These high incidence rates of anemia indicate a wide range of vitamin or mineral deficiencies but are primarily due to iron deficiency.

Data in our study suggest that the independent risk factors for anemia were female, lower hematocrit and hemoglobin levels, and microcytic anemia. However, SG and elevated BMI levels were the factors that likely decreased the risk of anemia postoperatively. These findings are comparable to the literature review by Bjørklund et al. [9]. Accordingly, their investigations disclosed that the factors linked to higher incidence rates of anemia were gender and the type of bariatric surgery, adding that females and RYGB were at increased risk of anemia for at least two times higher than males and other bariatric procedures. In a study conducted in Canada [6], an increased risk for iron deficiency was associated with malabsorptive procedures and low baseline ferritin, while younger age and low baseline ferritin were the risk factors for iron deficiency anemia (IDA). In our study, the prevalence of anemia according to age and the presence of comorbidities was not significantly different (p>0.05). On the other hand, two studies published in Canada [24] and the USA [25] suggested women with menstrual disorders, peptic ulcer disease, and longer duration of follow-up were at significant risk of developing anemia postoperatively. Previous reports indicated that females may have been at receiving end of developing anemia postoperatively and, therefore, may need extra attention to manage this nutritional deficiency effectively.

Moreover, we noted a significant decrease in BMI mean values before and after surgery, while a significant increase in the mean values of MCV has been detected. However, we found no significant variations in the mean values of hemoglobin, hematocrit, and vitamin B12 pre- and postoperatively. In Israel [7], comparing the outcome between One Anastomosis Gastric Bypass (OAGB) versus Sleeve Gastrectomy (SG), it was found that the OAGB group presented with a steep decline in total cholesterol and low-density lipoprotein (LDL), an increase in folate, and a greater decrease in hemoglobin and albumin than SG group. However, the results were insignificant in terms of vitamin B12, iron, and ferritin values. In Australia [10], 29% of the women presented with elevated homocysteine levels after bariatric surgery, and they also recorded low levels of ferritin (15%), RBC folate (12%), and vitamin B12 (11%). The authors suggested that routine nutrition screening and appropriate supplements are crucial in this population group due to the persistent deterioration of micronutrients.

According to reports, food supplements, medications, and IV drug therapies are the most effective treatment for iron deficiency [26-27]. In our study, most patients (97.7%) took multivitamins to treat nutritional deficiencies for at least three months (80.8%). In a systematic review published in Canada [24], based on 16 studies, prophylactic iron was the most commonly sought supplementation, and two studies indicated therapeutic iron supplementation intended for iron-deficient patients with varying dosages of 7 to 80 mg daily.

The limitation of the research is that it focuses on finding out the prevalence of anemia regardless of its type. Lack of availability of iron status markers, inflammatory markers and serum folate has limited the scope of this study to the prevalence of anemia in general without a clear differentiation between types of anemia after bariatric surgery in the studied sample. Unfortunately, a very high percentage of patients were lost to follow up. Only 30 out of 146 and 80 out of 374 are available for the BMI results.

## Conclusions

The prevalence of anemia among patients who underwent weight loss surgery was 28.1%. Independent risk factors for anemia were female gender, microcytic anemia, low hematocrit, and hemoglobin levels. However, sleeve gastrectomy and elevated BMI levels were assumed as the protective factors for developing anemia postoperatively. Anemia and other nutritional deficiencies are linked to complications after bariatric surgery. Effective treatment and monitoring are crucial in the management of patients post-bariatric surgery. Therefore, adherence to follow-up is needed to monitor nutritional deficiencies among this population group. Appropriate supplementation should also be monitored to combat anemia and improve patients' quality of life.
